# The NEIL Memory Research Unit: psychosocial, biological, physiological and lifestyle factors associated with healthy ageing: study protocol

**DOI:** 10.1186/s40359-015-0079-y

**Published:** 2015-06-27

**Authors:** Caoimhe Hannigan, Robert F. Coen, Brian A. Lawlor, Ian H. Robertson, Sabina Brennan

**Affiliations:** NEIL (NeuroEnhancement for Independent Lives), Trinity College Institute of Neuroscience, Trinity College, Dublin 2, Ireland; Mercers Institute for Research on Ageing, Hospital 4, St James’s Hospital, Dublin 8, Ireland

**Keywords:** Aging, Alzheimer’s disease, Cognitive decline, Cognition, Cognitive reserve, Cohort studies, Dementia, Independent living, Memory, Risk factors

## Abstract

**Background:**

Population ageing is a global phenomenon that has characterised demographic trends during the 20th and 21st century. The rapid growth in the proportion of older adults in the population, and resultant increase in the incidence of age-related cognitive decline, dementia and Alzheimer’s disease, brings significant social, economic and healthcare challenges. Decline in cognitive abilities represents the most profound threat to active and healthy ageing. Current evidence suggests that a significant proportion of cases of age-related cognitive decline and dementia may be preventable through the modification of risk factors including education, depressive symptomology, physical activity, social engagement and participation in cognitively stimulating activities. The NEIL Memory Research Unit cohort study was established to investigate factors related to brain health and the maintenance of cognitive function.

**Methods:**

A cohort of 1000 normally ageing adults aged 50 years and over are being recruited to participate in comprehensive assessments at baseline, and at follow-up once every 2 years. The assessment protocol comprises a comprehensive neuropsychological battery, some basic physical measures, psychosocial scales, questionnaire measures related to a range of health, lifestyle and behavioural factors, and a measure of resting state activity using electroencephalography (EEG).

**Discussion:**

The NEIL Memory Research Unit cohort study will address key questions about brain health and cognitive ageing in the population aged 50+, with a particular emphasis on the influence of potentially modifiable factors on cognitive outcomes. Analyses will be conducted with a focus on factors involved in the maintenance of cognitive function among older adults, and therefore will have the potential to contribute significant knowledge related to key questions within the field of cognitive ageing, and to inform the development of public health interventions aimed at preventing cognitive decline and promoting active and healthy ageing.

## Background

Population ageing is a global phenomenon that has characterised demographic trends during the 20th and 21st century, and presents both opportunities and challenges to society (Park & Reuter-Lorenz 2009). While an ageing population brings opportunities linked to the wealth of knowledge and experience possessed by older citizens; the increasing health, social and financial support needs of older adults place a significant societal burden in terms of healthcare and socio-economic provision. In almost all regions of the world, older adults represent the fastest growing proportion of the population, with the 60+ age group projected to be growing 3.5 times as rapidly as the total population by 2025–2030 (United Nations Population Division 2001). In Ireland, the proportion of the population aged 65+, which was stable at 11 % for the past 40 years, is predicted to reach 22 % by 2041 (McGill 2010; Layte 2009). Perhaps one of the most formidable challenges associated with an ageing population is the potential for considerably increased incidence of age-related cognitive impairment. Advancing age is the greatest risk factor for neurodegenerative disorders such as Alzheimer’s disease (AD) and other dementias (Mangialasche et al. 2012). The health and social cost of dementia disorders is considerable, with dementia care currently costing more than heart disease, stroke and cancer care combined. The current cost of dementia services per annum is estimated at €160 billion in Europe and €1.69 billion in Ireland. These estimates do not account for the concomitant psychological and social impact that dementia disorders have on individuals and caregivers.

Much of the cognitive decline experienced by older adults is not due to specific dementia pathologies (Henderson 2014); and normal, non-pathological ageing is associated with more subtle decline in a number of cognitive domains including executive functioning, speed of processing, memory, language and psychomotor ability (Buckner 2004). Age-related cognitive impairment that does not reach the threshold for dementia diagnosis is associated with reduced quality of life, increased health-care costs, increased neuropsychiatric symptoms, increased disability and increased risk for progression to dementia (Albert et al. 2002; Lyketsos et al. 2002; Edland et al. 2002).

Cognition is critical for mental and physical health, and social and emotional wellbeing. In turn, physical health, psychological health and degree of social engagement affect cognitive health. As treatments to delay onset and reduce incidence of heart disease, cancer and stroke become increasingly available, neurodegenerative conditions and cognitive decline are set to become one of the leading causes of mortality in an ageing population (Depp et al. 2012). Among the growing number of individuals aged 65+, the prospect of experiencing cognitive decline that results in a loss of independence is reported as one of the most feared aspects of the ageing process, and neurocognitive frailty is currently considered to be the greatest obstacle to successful, active and healthy ageing (Park & Reuter-Lorenz 2009; Daffner 2010).

The nature and severity of cognitive changes that occur with age are heterogeneous, ranging from essentially preserved functioning observed in individuals who are sometimes referred to as “super-elderly”, to the severe impairments observed in individuals diagnosed with dementing disorders (Daffner 2010; Anderson 2008). The different trajectories of cognitive decline observed among older adults are not related to one common process of “brain ageing”, but rather result from distinct cascades associated with non-pathological ageing and neurodegenerative disease states (Anderson 2008). The observation that some individuals live into old age with minimal decline, together with increasing evidence for brain plasticity in response to environment and experience across the lifespan, has sparked considerable global interest in understanding how older adults can maintain cognitive function. Preventing cognitive decline is crucial in order to extend independent living and promote active and healthy ageing. The identification of preventative strategies to maintain cognitive health can be considered a key priority for the reduction of age-associated disability and morbidity (Depp et al. 2012).

While further research is needed, current evidence – primarily from observational and epidemiological studies – suggests that a range of both genetic and environmental factors influence individual cognitive trajectories and cognitive decline during ageing (Mangialasche et al. 2012). It is likely that a multitude of factors contribute to the inter-individual differences observed in age-related cognitive outcomes. Potential risk and protective factors include ApoE status, midlife hypertension, depressive symptomology, education, socio-economic status, occupational attainment, dietary patterns, social engagement, participation in cognitively stimulating activities, and health behaviours such as physical activity and not smoking (Mangialasche et al. 2012; Stern 2012; Barnes & Yaffe 2011). It has been suggested that approximately half of all cases of Alzheimer’s disease worldwide may be attributable to known risk factors, a number of which are modifiable, raising the possibility that some of these cases may be preventable through risk factor modification (Yaffe et al. 2014). Prevention of cognitive decline and dementia is a legitimate, evidence based approach, and epidemiological research supports the possibility of reducing dementia prevalence and age-specific incidence through addressing modifiable risk factors (Cleary & McAvoy 2014). In an important review paper, Barnes & Yaffe (2011) suggested that a 10-25 % reduction in all of seven potentially modifiable risk factors – diabetes, midlife hypertension, midlife obesity, smoking, depression, cognitive inactivity or low educational attainment, and physical inactivity – could prevent up to 1.1 to 3 million AD cases worldwide.

A large number of longitudinal studies have been established to investigate the relationship of a range of factors to cognitive decline in older adults, including, for example, the Rush Memory and Ageing Project, the Nun Study, the Victoria Longitudinal Study, the Health and Retirement Study (HRS), the English Longitudinal Study of Ageing (ELSA) and the Maastricht Ageing Study. In addition to risk factors for cognitive decline and dementia, longitudinal studies can identify protective factors associated with the maintenance of cognitive function among participants considered to exhibit “successful ageing”. The identification of such risk and protective factors has the potential to inform public health interventions aimed at reducing disability, improving quality of life and decreasing social, healthcare and economic challenges associated with an ageing population.

The NEIL (Neuro-Enhancement for Independent Lives) Memory Research Unit was established to follow a large group of normally ageing adults in Ireland, in order to address key questions about factors involved in brain health and the maintenance of cognitive function in the face of age-related neural changes, with a particular focus on the impact of potentially modifiable risk and protective factors on cognitive trajectories among our ageing cohort. The study protocol was designed to include some parallel measures to The Irish Longitudinal Study of Ageing (TILDA) (TILDA 2010), a nationally representative study of adults aged 50+, in order to allow for comparability with national norms generated from the TILDA dataset. The Memory Research Unit protocol contains a more comprehensive cognitive battery than was feasible for inclusion in the TILDA study, and therefore allows for more detailed investigations of cognitive function in a similar population.

### Aims

The aims of the study are:

1. To establish a cohort of healthy older adults (aged 50+) who are willing to engage in research related to cognitive ageing on an ongoing basis.
2. To establish the cognitive profile of each participant at baseline through comprehensive neuropsychological evaluation, and to track changes in these cognitive profiles over time by means of repeat assessment.
3. To examine, in detail, cognitive trajectories and outcomes as the cohort ages and their associations with biological, social, lifestyle, behavioural and psychological factors. The longitudinal element of the study will focus on factors contributing to the maintenance of brain health and cognitive function in this age group.
4. To advance understanding of risk and protective factors related to cognitive ageing.

## Methods/Design

### Study design

**Fig. 1:**
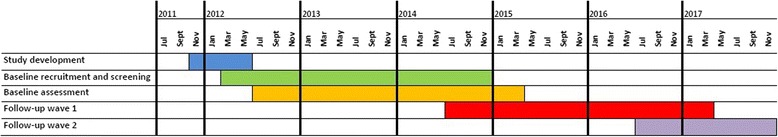
NEIL Memory Research Unit study timeline

**Fig. 2:**
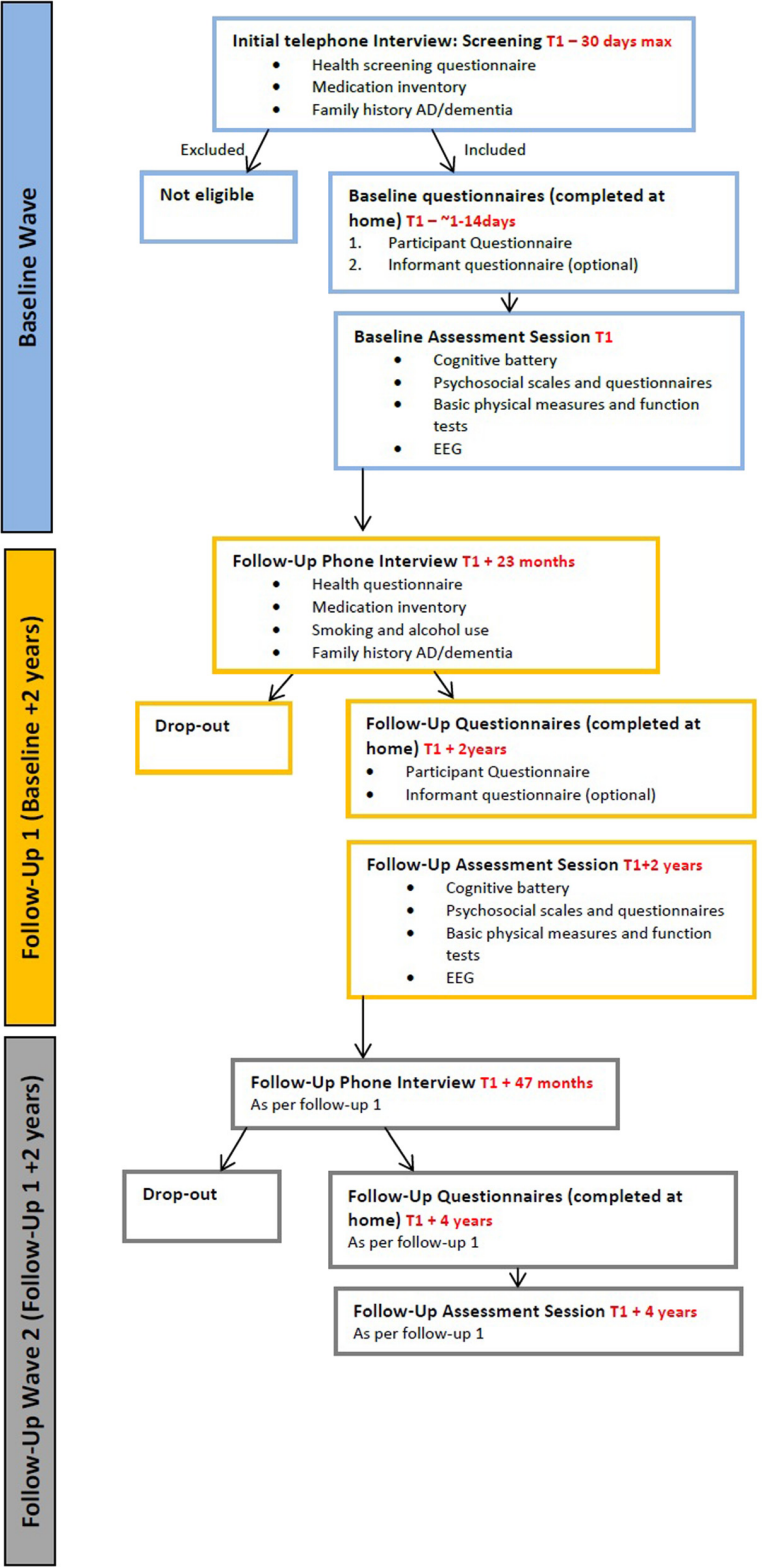
NEIL Memory Research Unit study design

We aim to recruit a total cohort of 1,000 participants aged 50 and over to this longitudinal observational study at baseline, and will invite these participants to complete follow-up assessments every 2 years. The study development, baseline and wave 1 follow-up phases of the study are currently supported using funds from a larger grant by the Atlantic Philanthropies, PhD Scholarships funded by the Irish Research Council, the Irish State-funded JobBridge internship scheme and voluntary hours undertaken by study investigators and associates. The study will continue until 2018, and we are actively seeking funding to extend the study duration and support additional waves of follow-up assessment beyond this date. The study plan involves a number of phases, including a study development period, recruitment phase, and baseline and follow-up assessment waves; and allows for overlap between waves in order to maximise the available resources and facilitate the greatest throughput of research participants. The current study schedule as proposed for the period 2011–2017 is illustrated in Fig. 1. A full list of the variables assessed and instruments used at each assessment wave is included in Table 1. The measures included in the protocol were selected through detailed literature reviews and consultation with a group clinical and academic ageing experts, in order to ensure the most appropriate measures were used for each construct. The study design is graphically represented in Fig. 2.

**Table 1:** Schedule of assessments and measures

		Screen/Phone Assessment	Baseline Questionnaire	Baseline Assessment	Follow Up Phone Assessment	Follow Up Questionnaires	Follow Up Assessment
Explain Study		X			X		
Obtain Consent		X					X
Inclusion and exclusion criteria		X					
*Health*							
Medical history	Health Screening Questionnaire (Christensen et al. 1992)	X					
	Self-report items	X			X		
Medication List	Self-report (verbatim from labels)	X			X		
Family history AD/dementia	Self-report item	X			X		
Self-rated health	Self-report items		X		X		
Frailty	Fried Frailty Index (Fried et al. 2001)			X			X
*Cognitive function*							
Self-rated memory	Self-report items		X			X	
Subjective memory complaints/ failures – self-rated	MAC-Q (Crook et al. 1992)					X	
	PRMQ (Smith et al. 2000)					X	
Informant/proxy rating of memory performance and complaints/failures	IQCODE (Jorm 1994)		X			X	
	Proxy PRMQ (P-PRMQ) (Smith et al. 2000)					X	
	Self-report items					X	
Reading ability/dyslexia screen	WRAT-3 Reading Test (Wilkenson 1993)			X			
Premorbid IQ	NART (Nelson 1982)			X			
Overall function	MMSE (Folstein et al. 1975)			X			X
	MoCA (Nasreddine et al. 2005)			X			X
Episodic Memory	WMS-IV Logical Memory Subtest (Weschler 2009)			X			X
	Bushke & Grober FCSRT (Grober & Buschke 1987)			X			X
	ACAD word and shape recognition task – immediate and delayed recall (Di Rosa et al. 2014)			X			X
Working Memory	WMS-III Letter Number Sequencing Subtest (Weschler 1997)			X			X
Prospective Memory	TILDA Experimental Task			X			X
Processing Speed	Colour Trails 1 (D’Elia et al. 1994)			X			X
	Choice Reaction Time experimental task (Brennan 2011)			X			X
Executive Function	Colour Trails 2 (D’Elia et al. 1994)			X			X
	Verbal (animal) fluency task			X			X
	CAMDEX Visual Reasoning Subtest (Roth et al. 1998)			X			X
Attention	Sustained Attention to Response (SART) experimental task (Robertson et al. 1997)			X			X
	ACAD Shapes Sustained Attention to Response (SSART) task (Di Rosa et al. 2014)			X			X
Visuo-Spatial	Landmark Spatial Bias Task (20 item) (Bellgrove et al. 2005)						X
*Demographics*							
Age	Self-report item	X	X		X	X	
Sex	Self-report item	X					
Marital Status	Self-report item		X			X	
Occupational Status – current	Self-report items		X			X	
Occupational History	Items adapted from Cognitive Reserve Index (Nucci et al. 2012)		X				
Educational attainment	Years of education, highest level completed – self report		X			X	
Caregiving	Self-report items		X			X	
*Behavioural/lifestyle*							
Smoking	Self-report items		X		X		
Alcohol Use	Self-report items		X		X		
	CAGE (Mayfield et al. 1974)		X		X		
Sleep Quality	Pittsburgh Sleep Quality Index (Buysse et al. 1989)		X			X	
	Stanford Sleepiness Scale(Hoddes et al. 1973)			X			X
Physical Activity	IPAQ – Short Form (Craig et al. 2003)			X			X
Leisure Activity/Cognitive Stimulating Activity	Leisure Activities Scale (House et al. 1982)		X			X	
	Lifetime Cognitive Activity Scale (Wilson et al. 2013)					X	
Boredom-proneness	Self-report item (Conroy et al. 2010)		X			X	
*Psychosocial*							
Self-rated mental health	Self-report item		X		X		
Depression	CES-D Scale (Radloff 1977)			X			X
Anxiety	HADS-A Anxiety subscale (Zigmond & Snaith 1983)			X			X
Perceived Stress	4-item Perceived Stress Scale (PSS-4) (Cohen et al. 1983)			X			X
Quality of Life/Life Satisfaction	CASP-12 (Wiggins et al. 2008)			X			X
	Self-report item			X			X
Social Connectedness	Berkman-Syme Social Network Index (Berkman & Syme 1979)			X			X
	Lubben Social Network Scale – 18-item (LSNS-18) (Lubben et al. 2003)					X	
*Physical*							
Height	Standing body height – Seca® stadiometer			X			X
Weight	Weight – Seca®electronic scale			X			X
Dominant hand	Self-report			X			X
Walking Speed	Timed walk – 16 ft (4.8768 m)			X			X
Grip Strength	Baseline® Hydraulic Hand Dynamometer – 2 measures per hand			X			X
*Physiological*							
EEG - Resting State	3 min eyes open, 3 min eyes closed			X			X

*ACAD* Automated Cognitive Assessment Delivery; *CAMDEX* Cambridge Mental Disorders of the Elderly Examination; *CES-D* Centre for Epidemiologic Studies Depression Scale; *FCSRT* Free and Cued Selective Reminding Test; *HADS-A* Hospital Anxiety and Depression Scale; *IPAQ* International Physical Activity Questionnaire

*IQCODE* Informant Questionnaire for Cognitive Decline in the Elderly; *LSNS-18* Lubben Social Network Scale (18 item); *MAC-Q* Memory Assessment Clinic Questionnaire; *MMSE* Mini-Mental State Examination; *MoCA* Montreal Cognitive Assessment; *NART* National Adult Reading Test; *PRMQ* Prospective Retrospective Memory Questionnaire; *PSS-4* Perceived Stress Scale (4 item); *SART* Sustained Attention to Response Task; *SSART* Shapes Sustained Attention to Response Task; *TILDA* The Irish Longitudinal Study of Ageing; *WMS* Weschler Memory Scale; *WRAT-3* Wide Range Achievement Test 3

To date, over 1,000 individuals have registered interest in taking part in the study, and 693 participants have completed baseline assessments. Baseline descriptive statistics for the sample tested to date are provided in Table 2. The first wave of follow-up testing began in July 2014, and as of December 2014 86 participants have been invited for follow-up assessment with a retention rate of 81.4 %.

**Table 2:** Descriptive statistics for current baseline sample

N	693
Age, years, mean (SD)	64.23 (6.92)
Age group	
50–59, %	26
60–69, %	54.1
70–79, %	17
80–89, %	2.9
Female, %	65.8
Education, years, mean (SD)	15.04 (3.45)
Education level	
Primary, %	3.9
Secondary, %	21.4
Tertiary, %	74.6
CES-D ≥16, %	8
Self-rated health	
Excellent, %	25.4
Very good, %	52.8
Good, %	18.0
Fair, %	3.1
Poor, %	0.7

### Ethical considerations

All study procedures are approved and authorised by the School of Psychology Research Ethics Committee (SPREC) at Trinity College Dublin. Written, informed consent is obtained from all participants, and participants are free to withdraw from the study at any time. Participants can also withdraw their data from the study at any point after they have completed assessments. All data is stored under a unique study ID code, without participant names or other identifying information. The study is non-invasive and imposes no significant risks.

### Participant recruitment and screening

The cohort is recruited from members of the general public in the Republic of Ireland who are aged over 50 years, in good health, and are in a position to attend Trinity College Dublin for an assessment session once every 2 years. Potential participants are provided with information about the study through advertisements, articles and interviews with the study investigators in local and national media (radio and print); community based information sessions provided by the study team; announcements in newsletters and circulars of relevant age-related organisations (for example Age Action Ireland, Active Retirement Ireland); through existing networks with ageing organisations; and at national conferences and events. Following receipt of this information, individuals can contact the study team in order to register their interest in taking part in the study.

Each potential participant receives a postal information pack that includes an introduction letter, a study information leaflet, consent forms and a stamped addressed envelope. Any individual who decides to take part in the study returns the signed consent forms by post, and is then contacted to complete an initial brief telephone interview. The purpose of this phone interview is two-fold: it is designed to collect information relating to history of a range of health conditions, family history of AD/dementia and a full list of the medications taken on a regular basis by the participant; and it also serves as a screening interview, with items included to specifically determine whether the participant meets any of the study exclusion criteria. The phone interview includes items from the Christensen Health Screening Questionnaire (Christensen et al. 1992) with some minor adaptations, along with additional items generated by the study investigators. An inventory of all current medications and long-term prescriptions are collected by asking the participant to read verbatim the name, dosage and frequency of each medication from the labels/boxes. The inclusion criteria imposed at baseline are: resident in the Republic of Ireland and in a position to travel to Trinity College for assessment, aged at least 50 years, and fluent in English to a standard sufficient for completion of neuropsychological assessment. Exclusion criteria are history of stroke, epilepsy, major psychiatric disorder, drug or alcohol abuse within the past 5 years, current use of anti-psychotic or anti-epileptic medication, self-report of significant memory problems or dementia, or problems with vision or hearing that would prevent neuropsychological evaluation.

### Baseline assessment

Following the initial telephone screening interview, all participants who do not meet any exclusion criteria are sent questionnaires to be completed at home prior to their assessment, and scheduled for a baseline assessment session. The postal questionnaire contains detailed demographic items along with scales to measure social, lifestyle and behavioural factors. Information collected in this questionnaire includes date of birth, marital status, occupational status and history, information about caregiving, self-rated health and memory, alcohol use and smoking behaviour, loneliness (De Jong & Van Tilburg 2006), sleep quality (Buysse et al. 1989), and participation in leisure activities (House et al. 1982). Participants are also sent a copy of the IQCODE (Informant Questionnaire for Cognitive Decline in the Elderly) (Jorm 1994), for a close relative or friend to complete.

Baseline assessment sessions take place at the NEIL Memory Research Unit in Trinity College Dublin. The assessment protocol entails a detailed assessment of cognitive function, psychosocial, behavioural, physical and physiological factors by means of:

### *a*) Cognitive assessment battery

Cognitive function is assessed using a 16 item battery of neuropsychological and experimental measures (see Table 1). The tests included in this battery were selected to provide measures of global cognition, along with functioning in a number of domains including episodic memory, working memory, prospective memory, executive function, speed, and attention. The WRAT-3 Reading subtest (Wilkenson 1993) was included as a screen for dyslexia or reading difficulties. The NART (National Adult Reading Test) (Nelson 1982) was included to provide an estimate of premorbid function.

### *b*) Psychosocial scales and questionnaires

Depressive symptoms, anxiety, perceived stress, life satisfaction, quality of life and social network are assessed using the scales detailed in Table 1. Physical activity was assessed using the Short Form of the International Physical Activity Questionnaire (Craig et al. 2003), which provides an objective measure of energy expenditure (MET-minutes per week) as well as a categorical measure of physical activity level (low, medium, high).

### *c*) Basic physical measures and function tests

Standing body height (cm) is measured using a stadiometer (Seca® - 216). Weight (kg) is evaluated with an electronic scale (Seca® - 876). Hand-grip strength (kg) is measured using a Baseline® Hydraulic Hand Dynamometer (Standard), with two readings taken from each hand. Walking speed (s/ms) is measured over a distance of 16 ft (4.8768 m). Frailty classifications are generated for each participant using the Fried Index (Fried et al. 2001).

### d) Electroencephalogram

Electroencephalography is used to collect a measure of resting state brain activity. EEG measures were included in order to collect direct measures of brain activity without the expense or necessary exclusions of methods such as MRI. The EEG data will be used in order to investigate potential electrophysiological markers of age-related cognitive decline. Spectral analysis of EEG recordings have proved to be promising potential biomarkers of cognitive deficits in recent studies (Moretti et al. 2013). EEG signals are recorded using an ActiveTwo system (BioSemi, The Netherlands) from 32 surface electrodes. EEG recordings are collected while the participant sits at rest with their eyes closed for 3 minutes, and their eyes open for 3 minutes.

All baseline measures are collected during one assessment session lasting approximately 2.5-3 hours including regular breaks to avoid participant fatigue. All research sessions take place at either 10am or 2pm, in order to allow for statistical control for time of day effects. All measures are administered in the same order to each participant, using strict standard operating procedures and scripts to ensure consistency of the testing process. Data is entered directly to an automated, custom-built interface via a laptop computer during testing.

The full protocol was tested in a pilot study (n = 20) and shown to be fully practical and practicable. The schedule was shown to be acceptable to participants. Based on feedback from pilot participants, a number of grammatical and structural changes were made to the postal questionnaire in order to improve readability and ease of completion.

### Follow-up assessments

All participants are invited to complete a follow-up assessment every 2 years. Assessment procedures will be identical for each follow-up wave. A full list of variables assessed and instruments used during follow-up waves is detailed in Table 1. Follow-up assessments follow a similar structure to the baseline evaluation – consisting of a phone interview, questionnaires to be completed by the participant and an informant at home, and one assessment session at Trinity College Dublin.

During the follow-up phone interview, information is collected about significant health changes since baseline assessment, health conditions, family history of AD/dementia, and smoking and alcohol use. Any participant who reports a change in their smoking behaviour or alcohol use are asked to provide more detailed information by repeating measures related to these variables that were administered at baseline. An inventory of medications is again collected using the identical procedures to baseline assessment.

The postal questionnaire completed by participants at follow-up repeats all items from the baseline questionnaire, with the exception of occupational history, in order to allow for assessment of change in these factors over time. The original baseline questionnaire items are supplemented with additional items and scales designed to collect more detailed information about subjective memory ratings, complaints (Crook et al. 1992) and failures (Smith et al. 2000); along with an additional social network scale (Lubben et al. 2003), and an additional activities scale that assesses participation in cognitively-stimulating activities across the lifespan (Wilson et al. 2013). Participants are again sent an informant questionnaire, to be completed by a close friend or relative, at each follow-up assessment point. This questionnaire repeats the IQCODE (Jorm 1994) from baseline, and is supplemented with additional items designed to assess subjective ratings of the participant’s memory performance (compared to others their age, change since their last assessment, decline in memory), and with the informant version of the Prospective Retrospective Memory Questionnaire (PRMQ) (Smith et al. 2000).

The follow-up assessment session follows an almost identical protocol to the baseline assessment, repeating all measures with the exception of the National Adult Reading Test and the WRAT-3 Reading sub-test. One additional task designed to measure spatial bias is included at the end of the cognitive battery during follow-up assessments. All measures are again collected during one assessment session, lasting approximately 2.5-3 hours including breaks.

### Sample size considerations

Following protocol design from previous studies of similar nature (e.g.(Collerton et al. 2007)), we considered formal sample size calculations for the study as a whole to be unfeasible, given that there are a large number of specific factors to be analysed. The target sample size was selected based on a number of considerations, including sample sizes used in previous studies of a similar design which proved sufficient for a range of statistically significant conclusions to be drawn. The sample size was also selected based on pilot recruitment and testing, to account for the number of participants that could feasibly be recruited and tested per year with the resources available.

Table 3 details the age distribution of the population of Ireland aged 50+, based on data from the 2011 Census (Central Statistics Office 2011), which can be used to provide some indication of the expected age distribution of our sample. At the time of the 2011 Census, 27.75 % of the Irish population was aged over 50 years. The age distribution of our baseline sample recruited to date (see Table 2) shows that in our current sample, participants aged 60–69 are over-represented, and participants aged 50–59 and 80+ are under-represented. The proportion of participants aged 70–79 is largely in line with what would be expected based on the Census data in Table 1. We will attempt to address these issues in future recruitment, perhaps by targeting recruitment activity towards age groups that are currently under-represented. These issues may also be addressed with statistical weighting in future analyses.

**Table 3:** Age distribution of Irish population aged 50+ in 2011 Census^a^

Age group	N	% of population aged 50+
50-59	518,908	40.76
60-69	392,424	30.82
70-79	233,226	18.32
80+	128,529	10.10
Total population aged 50+	1,273,087	
Total population	4,588,252	

^a^Data for these calculations was taken from the “Profile 2: Older and Younger” report of the 2011 Census of Ireland (Central Statistics Office 2011)

While statistical consideration of attrition was not feasible, attrition rates from a number of nationally representative longitudinal ageing studies provide some indication of the level of attrition that might be expected in our study. For example, among participants aged 55–64 years, 88 % of participants in the HRS, and 68 % of participants in ELSA responded to all three assessment waves in the period 2002–2006 (Banks et al. 2011). For participants aged 70–80 years, the percentage who responded to all three waves during the same period was 78 % in the HRS and 63 % in ELSA (Banks et al. 2011). Preliminary data on attrition rates TILDA, which completes follow-up assessments every 2 years, shows a total response rate of 86 % at wave 2 (Nolan et al. 2014).

While acknowledging that our current estimations are based on a small number of participants, the retention rate currently observed for our first wave of follow-up is largely in line with that of TILDA and is encouraging in terms of potential impact of attrition rates.

### Planned statistical analyses

Interim analyses will take place after the completion of each study phase. Descriptive analyses based on means, standard deviations, percentages, relative risks and 95 % confidence intervals will be used to describe the studied population. Given the comprehensive nature of the dataset collected, a wide range of analyses will be conducted to investigate individual research questions using subsets of the variables available, and specific analysis plans will be generated for each research question of interest. These specific analysis plans are beyond the scope of this paper and will be described fully in subsequent publications.

Upon completion of baseline assessment, cross-sectional analyses will be conducted to explore relationships between various factors measured and cognitive performance among the cohort. These cross sectional analyses will include logistic regression models and structural equation modelling techniques. As follow up data becomes available full analysis of the data will be conducted using multivariate statistical methods in order to model the effects of a range of predictor variables on cognitive trajectories. When data is available for 2 time-points, these analyses will include, for example, mixed models analyses and weighted analysis of covariance models with appropriate covariates of baseline measures and age and gender. Once data becomes available for 3 time-points, methods including linear growth curve modelling will be employed. Relevant covariates will be included as interaction terms.

Specific analysis plans will be designed to include statistical consideration of methodological issues related to longitudinal data collection, including missing data, practice effects, regression to the mean and attrition. For example, an attrition weight (inverse-probability) based on drop out from follow-up wave 1, using key variables of interest such as age, comorbidities and frailty, will be calculated and applied to subsequent analyses. In dealing with missing data, we intend to use methods including multiple imputation and Full Information Maximum Likelihood (FIML) estimation, depending on the specific analysis plan for the research question under investigation.

The primary outcomes of interest will be cognitive function measured at baseline, and change in cognitive function measured longitudinally. To limit the number of dependent variables and improve robustness of underlying cognitive constructs, raw test scores will be converted into Z scores using baseline sample means and standard deviations, and the average of these Z scores will be used to create a measure of global cognitive functioning. The measures will also be grouped into domains (e.g. episodic memory, executive function, attention, processing speed), and the average Z score for measures included in each domain will be calculated as a composite measure. The grouping of measures into domains will be guided by previous literature and exploratory factor analysis of our data. In the primary analysis, if data is available for the majority of tests that make up a domain score (e.g. 3 out of 5, or 2 out of 3 tests), data will be combined as described above and included. A sensitivity analysis including only cases with complete data for all cognitive measures will be conducted to investigate the impact of missing values.

### Quality control

#### Standardisation and training

The study is managed by a core team of senior investigators, and data collection is conducted by a team of research assistants who are recruited via state-funded internship schemes, volunteer psychology graduates and postgraduate students in the School of Psychology at Trinity College Dublin. Given that neuropsychological assessment and data collection will be completed by a team of research assistants, standardisation of protocols and rigorous training are a critical priority in order to ensure the validity of the data collected. Strict standard operating procedures for all data collection and input activities have been developed, including a detailed testing manual with scripts and instructions for the administration of all measures. All research assistants complete a comprehensive training program before beginning data collection, including a one day training course, practice research sessions, and a validation session where they are approved for testing by a senior investigator.

#### Data entry and processing - control, traceability and validation of results

All study data is recorded via a custom-built, automated, secure web-based system, following a strict standard operating procedure. This system was designed to reduce the level of human error in raw data entry – the user-friendly interface only allows values that lie within the correct possible range to be entered for each measure, and includes inbuilt checks for data completeness and validity – for instance, pop-up windows to remind the researcher of the correct time lag for delayed recall tasks, or to notify the researcher if any data is missing before saving. Encoding and initial processing of the collected data is automatically conducted by this system, which was designed, developed and rigorously tested by Trinity Centre for High Performance Computing (TCHPC) in collaboration with the senior investigators. The data entry system retains revision logs in order to allow traceability. Data is then exported from this system to .csv files for further processing and analysis – all steps taken to further process, clean or recode data are verified by two or even three people involved at specific stages of the procedure.

#### Follow-up and participant retention

In order to encourage continued participation and involvement with the study, participants are sent quarterly newsletters containing an update on study progress, along with information about other activities within our larger research program. They are also invited to events organised by the NEIL programme, such as Brain Health Awareness evenings or information talks. All enrolled participants receive a postal information pack every two years, inviting them to return for a follow-up assessment. One week after this information pack is sent, a research assistant contacts the participant by phone to discuss the information they have received, re-explain the study and arrange a follow-up appointment. Participants are asked to sign a form confirming their continuing consent to take part in the study before each follow-up assessment.

### Dissemination

Results will be disseminated at regional, national and international research conferences; in reports published by our research programme and in articles published in international peer-reviewed journals.

## Discussion

The NEIL Memory Research Unit cohort study will address key questions about brain health and cognitive ageing in the Irish population aged 50+, with a particular focus on the influence of potentially modifiable factors – such as physical activity, cognitive reserve, psychological health, social engagement or cognitively stimulating activities – on cognitive outcomes among an ageing population. The comprehensive nature of the data collected will allow for investigation of a range of key questions related to cognitive ageing. One of the main objectives of the study is to identify factors that are associated with different cognitive trajectories experienced by older adults. Regular follow-up assessments will allow for identification of this variability in individual cognitive trajectories among our cohort, and examination of their associations with a range of health, behavioural, psychosocial and lifestyle factors. Analyses will be conducted with a focus on factors involved in the *maintenance* of cognitive function among older adults, and therefore will have the potential to inform future intervention studies.

The study design has a number of strengths. Detailed neuropsychological evaluation of participants at each time point will facilitate exploration of specific cognitive profiles, and of the relationship of these profiles to a range of outcomes and predictors. Rigorous quality control procedures incorporated within the study protocol from the outset, including detailed standard operating procedures and automated systems to reduce error in data entry and processing, will ensure that the dataset produced is of high quality and validity. Published and highly-cited measures with established reliability and validity were selected whenever possible.

Given that our sample is self-selecting, it is likely to be biased towards high-functioning, well-educated, motivated volunteers – as is the case in many studies of cognitive ageing (Nebes et al. 2006). Preliminary descriptive analysis shows that a large majority of the sample recruited to date have completed third level education, suggesting that our total sample will have a disproportionate number of highly educated participants, which may limit the generalizability of our results to less-educated populations. Evidence from the cognitive reserve literature suggests that education may act as a ‘buffer’ against cognitive impairment, allowing highly educated individuals to tolerate a greater level of neuropathology before experiencing clinical symptoms of dementia (Stern 2012). As such, highly educated older adults may represent a uniquely ‘at risk’ group, given that subtle early cognitive decline may be difficult to detect using traditional norm-based neuropsychological approaches, and that the ‘window of opportunity’ for intervention may have passed by the time clinical impairment is detected. We therefore consider them to be a group of particular interest in terms of our research objectives, while acknowledging the issues of generalizability within our sample. We have included detailed measures of a range of cognitive reserve indicators within our study protocol that will permit comprehensive investigation of factors that contribute to reserve across the lifespan, and allow us to model the effects of cognitive reserve using multiple proxy indicators. This has not been possible in other studies that have used education as a single indicator to represent cognitive reserve.

A further issue related to a self-selecting sample is the over-representation of individuals between 60 and 69 years in the distribution of participants recruited to date, which again may limit the generalizability of the study results and may also have an impact in terms of the length of follow up required to detect cognitive change. We may address this issue with statistical weighting in future analyses, and also by targeting future recruitment activities towards under-represented age groups.

The use of a self-report medical history and relatively minimal exclusion criteria may also result in a sample including participants with health conditions that are not severe enough to prevent their participation in the study, but are known risk factors for neuropathology and likely to affect cognitive performance (Nebes et al. 2006). Indeed, there is considerable debate in the literature as to whether exclusion criteria for studies of older adults should be designed for the selection of a perfectly healthy sample, which could be described as ‘super-elderly’; or one that is more representative of a general population (Tisserand & Jolles 2003). Having considered this literature, our preference was for a sample more representative of the normally ageing population of Ireland aged 50+, many of whom will have health conditions or engage in behaviours that could influence cognition. Thus, our criteria were selected to achieve a balance between importance and practicality, excluding participants who had medical conditions or were taking medications that would be expected to have a significant impact on cognitive performance, without excluding an inordinate proportion of the population.

Unfortunately due to resource limitations, it was not possible for us to include collection of some potentially useful data types, such as blood chemistry, genetic measures or scanning, in the design of this study. These measures have been included in a number of previous longitudinal studies, and certainly would have added considerable value to our dataset had their inclusion been feasible. We acknowledge that the absence of these data types represents a limitation of our study. Given that one of the aims of this project was to establish a cohort of older adults willing to engage in cognitive ageing research on an ongoing basis, we included within our consent procedures an ‘opt in’ for participants who would be interested in being contacted to take part in further add-on studies from time to time. This provision has allowed for the possibility of collecting additional useful measures such as blood chemistry or neuroimaging, from at least a subset of our sample should funding become available to do so, and to analyse this additional data along with the data currently collected as part of the design of the main study detailed here.

This study will generate new knowledge to increase our understanding of cognitive ageing adding to data that has emerged from large longitudinal studies across the globe. As stated above, the study runs in parallel with TILDA, and the protocol contains a number of measures that are common to both studies. The characteristics of our self-selecting sample suggest that it is likely to contain a higher proportion of successfully ageing individuals with high cognitive reserve than the TILDA sample. Comparisons of outcomes among these two samples may add to knowledge relating to the effects of cognitive reserve, the efficacy of neuropsychological assessments in identifying cognitive decline among high performers as compared with the general population, and the potential risks associated with under-detection of cognitive decline among high performing individuals. In addition, the design of our study allows us to run sub-studies to collect additional data types from our participants, which may allow for investigation of additional research questions or interventions based on findings that emerge from the TILDA study.

Our study protocol contains measures of a number of variables that were identified, in a comprehensive review of longitudinal studies of ageing, as important for future investigation (Stanziano et al. 2010). For example, it was suggested that BMI and gait speed may emerge as an important index for prediction of health status and mortality (Stanziano et al. 2010), and the inclusion of these measures in our protocol will allow this potential index to be investigated as a predictor of cognitive decline. The authors also noted that while factors associated with socioeconomic status are routinely measured in longitudinal ageing studies, it remains unclear whether these factors are associated with functional status, cognitive outcomes and mortality (Stanziano et al. 2010). Our protocol contains detailed measures of occupational status and educational history across the lifespan, which will be investigated in relation to cognitive outcomes.

The identification of factors involved in the maintenance of cognitive health, with a view to developing public health intervention approaches, is a key priority for research aiming to address social, healthcare and economic challenges associated with demographic ageing. We believe that the data collected as part of this study has the potential to contribute important knowledge that is required to answer fundamental questions related to cognitive ageing, which will have major long-term relevance for the health of our rapidly ageing population.
